# Intergenerational hyperglycemia through epigenetic alterations of gametes

**DOI:** 10.1093/lifemeta/loac007

**Published:** 2022-06-16

**Authors:** Fei Mao, Xiaoying Li

**Affiliations:** Ministry of Education Key Laboratory of Metabolism and Molecular Medicine, Department of Endocrinology and Metabolism, Zhongshan Hospital, Fudan University, Shanghai 200032, China; Ministry of Education Key Laboratory of Metabolism and Molecular Medicine, Department of Endocrinology and Metabolism, Zhongshan Hospital, Fudan University, Shanghai 200032, China; Shanghai Key Laboratory of Metabolic Remodeling and Health, Institute of Metabolism and Integrative Biology, Fudan University, Shanghai 200438, China

**In a recent study published in *Nature*, Chen *et al.* reported a mechanism through which hyperglycemia is transmitted from female mice to their offspring through their eggs and DNA methylation of the paternal-derived pancreatic *Gck* promoter is increased due to a decrease of maternal effector TET3 dioxygenase.**

It is well established that diabetes can be inherited from parents to offspring. Although some allele variants have been identified to be associated with hyperglycemia, gene–environment interactions play a major role in mediating hyperglycemia. It has been a controversial topic about inheritance of acquired characteristics, and the Lamarckian inheritance has long been dismissed. To date, increasing evidence suggests that certain acquired characteristics can be passed on to next generation, and maternal and paternal inductions of intergenerational responses are indispensable. Paternal effects on hyperglycemia in offspring have been widely reported, including paternal low-dose streptozotocin (STZ)-induced hyperglycemia [1], chronic high-fat diet [2], and psychological stress-induced hyperglycemia [3]. Maternal effect on germ cells, however, remains largely unknown owing to the difficulties in discerning it from direct impact of *in utero* exposure.

A recent study published in *Nature* [4] revealed a mechanism through which high blood sugar is transmitted from female mice to their offspring through their eggs. First, the authors treated female mice with STZ to induce chronic hyperglycemia. They then fertilized the eggs from these mice *in vitro* using sperm from healthy males, and implanted them into healthy surrogate mothers, enabling them to exclude general effects caused by hyperglycemia in pregnancy, thereby focusing on the effect of hyperglycemia on the eggs *per se*. Notably, their offspring presented glucose intolerance, which is caused by deficient glucose-stimulated insulin secretion from pancreatic β cells. Further RNA sequencing analysis on hyperglycaemia (HG) MII oocytes revealed that decreased expression of maternal effector TET3 dioxygenase under hyperglycemia condition accounted for these observations. They found that oxidation of methylated DNA was markedly reduced in the paternal genome of fertilized eggs derived from the hyperglycemic mice than in those derived from the control group. The response of maternal TET3 to glucose level in oocytes has been rigorously proved through *in vitro* and *in vivo* study, as well as in pregnant women with diabetes. A previous study reported that oocyte-derived TET3 dioxygenase was primarily responsible for the oxidative modification of the cytosine methyl group of paternal DNAs in fertilized embryos, thereby initiating DNA demethylation of sperm-borne DNA and reprogramming throughout development [5]. Based on this finding, genome-wide methylation sequencing was performed on embryonic pancreatic islets in the offspring. The results showed that genes with hypermethylated promoter regions were mainly enriched in the insulin secretion pathway, including a promoter that drives pancreatic expression of the Glucokinase gene (*Gck*) that encodes the rate-limiting enzyme for glucose-stimulated insulin secretion. The authors went on to prove increased DNA methylation of the paternal-derived pancreatic *Gck* promoter at various stages of development, including postnatal phase. This hypermethylation was associated with reduced *Gck* expression in pancreatic islets. These results all suggested that in the progeny of female mice with hyperglycemia, the hypermethylation of insulin secretion genes (i.e. *Gck*) originated from the male pronucleus of fertilized eggs and persisted into the adult. The authors also reported increased level of methylation at the *Gck* promoter in early-stage embryos from a woman with diabetes compared with two healthy women, providing strong supporting evidence from a clinical perspective. Together, these data support the paternally derived *Gck* gene as an important target of maternal TET3. To further verify whether reduced TET3 expression alone contributes to *Gck* hypermethylation and glucose intolerance in the progeny from hyperglycemic eggs *in vivo*, the authors genetically engineered oocytes with TET3 depletion to mimic the effect of maternal hyperglycemia, impaired insulin secretion and glucose intolerance. *Vice versa*, rescue experiment by injecting TET3 mRNA into embryos improved glucose metabolism in offspring, whereas injecting catalytically inactive TET3 had no such an effect. Together, all these data suggest that paternal pronuclear DNA hypermethylation originated from abnormal TET3 expression in oocytes leads to metabolic dysfunction in progeny.

**Figure 1:**
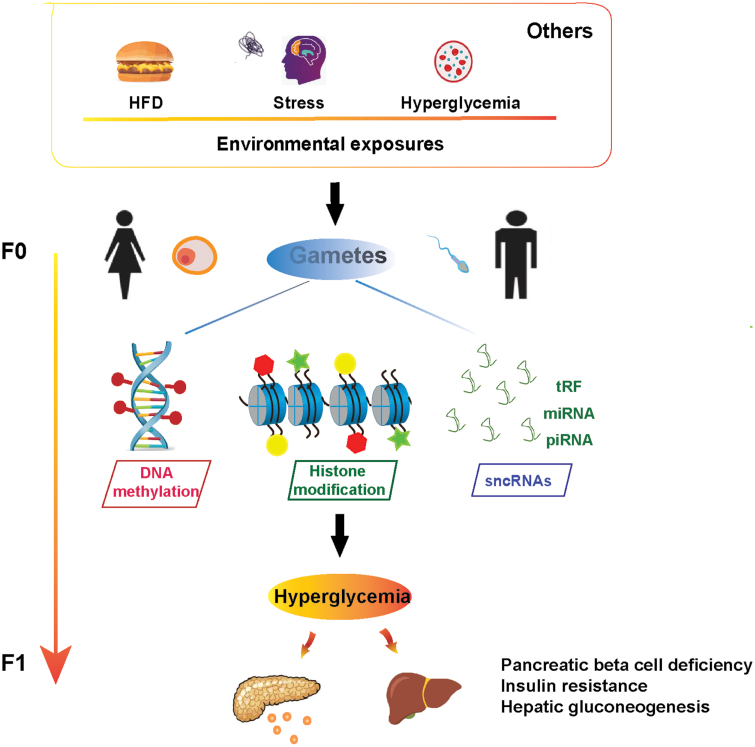
Intergenerational hyperglycemia through epigenetic alterations of gametes. Adverse exposures of high-fat diet, psychological stress, hyperglycemia, etc. lead to epigenetic changes of DNA methylation, histone modification, and sncRNAs in germ cells and consequently result in disorder of glucose metabolism in offspring.

Emerging evidence shows that epigenetic alterations, such as altered DNA methylation and histone modifications, as well as expression of small noncoding RNAs [6], can be inherited from parental gametes (sperm, eggs) to the organs of offspring (also called inter/transgenerational effect), and therefore affects metabolic features of the offspring, including hyperglycemia (Fig. 1). In line with this, a study from our group also reported that paternal psychological stress could influence glucose metabolism in offspring mice through increasing the methylation of Sfmbt2 promoter in sperm, which in turn led to downregulation of miR-466b-3p expression in the liver and increased hepatic gluconeogenesis [3]. All these studies clearly demonstrate that environmental exposures can influence metabolism characteristics in offspring through both maternal and paternal germlines. There are certain critical windows during which a specific stress may cause long-lasting consequences on different organs in later life of offspring. To delineate mechanisms responsible for intergenerational transmission of phenotypes, how and when epigenetic perturbations occurring in gametes influencing offspring need further study. Herein, our proposals for future investigation of intergenerational transmission of metabolic characters are: (i) How does the environmental input exactly affect the germ cell epigenome? Do hormones and nutrient signaling involve in this process? (ii) How are the epigenetic makers in gametes reprogramed in the organs of offspring? (iii) Does epigenetic alteration in gametes influence certain period of development like preimplantation embryonic development, later-stage postimplantation, or tissue-specific stem cells remodeling? (iv) What else parental characters can be transmitted through gametes apart from diets, stress, and those which have already been reported? For the complexity of this issue, the big-data strategy at genomic and epigenomic levels might be an ideal approach to find the answers.
