# Correction to: Intergenerational hyperglycemia through epigenetic alterations of gametes

**DOI:** 10.1093/lifemeta/load023

**Published:** 2023-06-16

This is a correction to: Fei Mao, Xiaoying Li, Intergenerational hyperglycemia through epigenetic alterations of gametes, *Life Metabolism*, Volume 1, Issue 2, October 2022, Pages 107–108, https://doi.org/10.1093/lifemeta/loac007

In the originally published version of this manuscript there was an omission in the conflicts of interest declaration. The declaration has been changed to the following to acknowledge Xiaoying Li’s role as an Associate Editor of *Life Metabolism*: “The authors declare no conflict of interest exists, except Xiaoying Li, one of the authors, is also an Associate Editor of *Life Metabolism*, and he was blinded from reviewing or making decisions on the manuscript.”

This error has been corrected.

